# Correction: Ma et al. Rad52 Oligomeric N-Terminal Domain Stabilizes Rad51 Nucleoprotein Filaments and Contributes to Their Protection against Srs2. *Cells* 2021, *10*, 1467

**DOI:** 10.3390/cells10113207

**Published:** 2021-11-17

**Authors:** Emilie Ma, Laurent Maloisel, Léa Le Falher, Raphaël Guérois, Eric Coïc

**Affiliations:** 1 Université de Paris and Université Paris-Saclay, Inserm, LGRM/iRCM/IBFJ-CEA, UMR Stabilité Génétique Cellules Souches et Radiations, F-92265 Fontenay-Aux-Roses, France; emilie.ma@cea.fr (E.M.); laurent.maloisel@cea.fr (L.M.); 2 Present address: Precision Oncology Genomics, Oncology Therapeutic Area, Sanofi R&D, F-94403 Vitry-Sur-Seine, France; lea.lefalher@live.fr; 3 Université Paris Saclay, CNRS, LBSR/i2BC-CEA, Institute for Integrative Biology of the Cell (I2BC), F-91198 Gif-Sur-Yvette, France; raphael.guerois@cea.fr

The authors wish to make the following changes to their paper [1]. The Figure 5 should be changed from:

**Figure 5:**
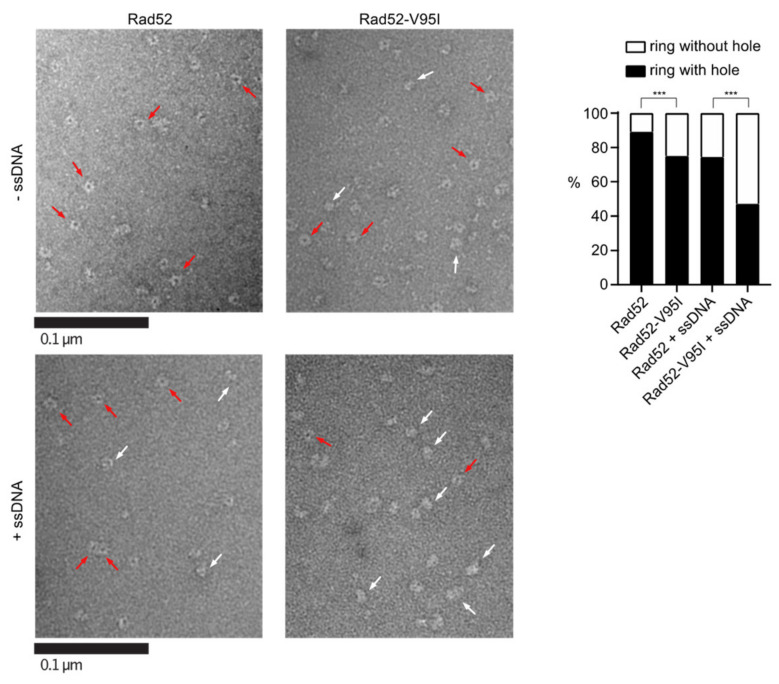
EM analysis of Rad52 and Rad52-V95I without DNA (upper panels) or with 400nt-ssDNA (lower panels). All experiments were performed with FLAG-tagged Rad52 proteins. The protein-ssDNA complex was formed at a ratio of 1:20 nucleotides. Some representative rings with hole (red arrows) and without hole (white arrows) are shown. The percentage of each molecular species is plotted on the histogram (right panel). Two independent experiments were performed with very similar results. The results of the individual biological replicates were pooled (933 molecules analyzed); *** *p* < 0.001 (two-tailed Fisher’s exact test).

to:

**Figure 5:**
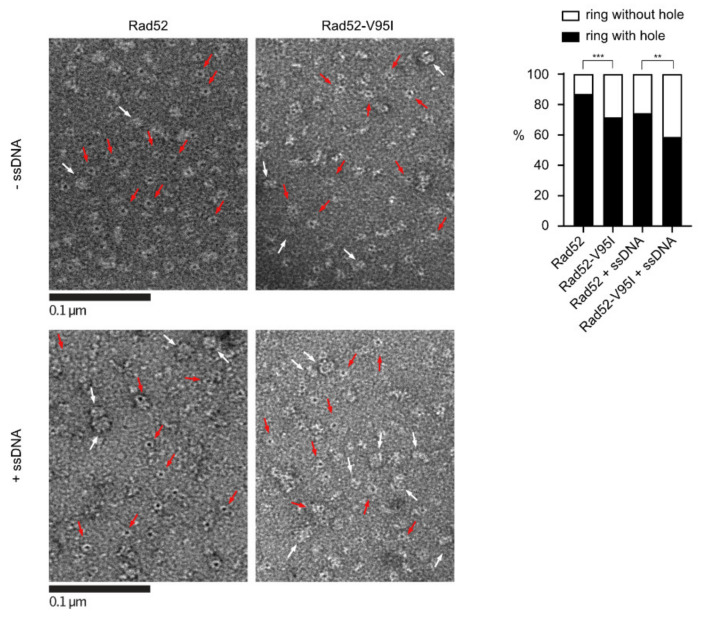
EM analysis of Rad52 and Rad52-V95I without DNA (upper panels) or with 400nt-ssDNA (lower panels). All experiments were performed with FLAG-tagged Rad52 proteins. The protein-ssDNA complex was formed at a ratio of 1:20 nucleotides. Some representative rings with holes (red arrows) and without holes (white arrows) are shown. The percentage of each molecular species is plotted on the histogram (right panel). 641 molecules were analyzed; *** *p* < 0.001; ** *p* < 0.05 (two-tailed Fisher’s exact test).

Regard this correction, the other parts of the manuscript related to this figure also need to be modified.

In Section 2.12, Electron Microscopy Analysis, “For ring analysis, Rad52 was diluted to 0.27 µM in 10 mM Tris pH 7.5, 50 mM KCl, 2 mM MgCl_2_ and 1 mM DTT. A fraction of the dilution was deposited onto a 400 mesh copper grid coated with a thin carbon film, previously activated by glow-discharge in the presence of pentylamine (Sigma-Aldrich, Merck, St. Quentin Fallavier, France). After 1 min, grids were colored with aqueous 2% (*w*/*v*) uranyl acetate (Sigma-Aldrich, Merck, St. Quentin Fallavier, France) and then dried with ashless filter paper (VWR Fontenay-sous-bois, France). Observations were carried out using a Thermo fisher TECNAI 12 transmission electron microscope in filtered annular dark field mode. Images were acquired with a Veletta digital camera and the iTEM software (Olympus, Soft Imaging Solutions Rungis, France).” should be changed to “For ring analysis, Rad52 was diluted to 2.7 μM in 10 mM Tris pH 7.5, 50 mM KCl, 2 mM MgCl_2_, and 1 mM DTT. Samples were analyzed by conventional electron microscopy using the negative staining method. Three microliters of sample suspension were deposited on an air glow-discharged 400 mesh copper carbon-coated grid for 1 min. The excess liquid was blotted, and the grid rinsed with 2% *w*/*v* aqueous uranyl acetate. The grids were visualized at 100 kV with a Tecnai 12 Spirit transmission electron microscope (Thermo Fisher, New York, NY, USA) equipped with a K2 Base 4k × 4k camera (Gatan, Pleasanton, CA, USA).”

“Reactions were diluted 10 times and 5 µL of the dilution was deposited onto a 400 mesh copper grid coated with a thin carbon film, previously activated by glow-discharge in the presence of pentylamine (Sigma-Aldrich, Merck, St. Quentin Fallavier, France). After 1 min, grids were colored with aqueous 2% (*w*/*v*) uranyl acetate (Merck, France) and then dried with ashless filter paper (VWR Fontenay-sous-bois, France). Observations were carried out using a Thermo Fisher TECNAI 12 transmission electron microscope. Images were acquired with a Veletta digital camera and the iTEM software (Olympus, Soft Imaging Solutions Rungis, France).” should be changed to “Samples were analyzed by conventional electron microscopy using the negative staining method. Five microliters of sample suspension were deposited on an air glow-discharged 400 mesh copper carbon-coated grid for 1 min. The excess liquid was blotted, and the grid rinsed with 2% *w*/*v* aqueous uranyl acetate. The grids were visualized at 100 kV with a Tecnai 12 Spirit transmission electron microscope (Thermo Fisher, New York, NY, USA) equipped with a K2 Base 4k × 4k camera (Gatan, Pleasanton, CA, USA).”

In Section 3.4, “With WT Rad52, 90% of the structures observed were well-shaped rings displaying a distinctive hole in the middle. Conversely, with Rad52-V95I only 75% of rings had a hole, confirming that this mutation slightly destabilizes the ring organization. Surprisingly, we obtained similar results by incubating Rad52 with ssDNA. This effect was more pronounced when we added ssDNA to Rad52-V95I (only 47% of well-shaped rings).” should be changed to “With WT Rad52, 87% of the structures observed were well-shaped rings displaying a distinctive hole in the middle. Conversely, with Rad52-V95I, only 72% of rings had a hole, confirming that this mutation slightly destabilizes the ring organization. Surprisingly, we obtained similar results by incubating Rad52 with ssDNA. This effect was more pronounced when we added ssDNA to Rad52-V95I (only 58% of well-shaped rings).”

In the Acknowledgments, “We also thank Pauline Dupaigne and Eric Le Cam (UMR 9019, IGR, Villejuif) for their help with TEM.” should be removed. The Acknowledgments part should be: This work benefited from the CryoEM platform of I2BC, supported by the French Infrastructure for Integrated Structural Biology (FRISBI) (ANR-10-INSB-05-05) and member of IBISA. We are especially grateful to Malika Ould Ali for her help with TEM. We thank the members of the molecular biology platform (Cigex) of our institute, Xavier Veaute and Didier Busso for cloning, directed mutagenesis, and for RPA, Rad51, and Srs2 purification. We are grateful to Vincent Géli for the gift of RPA antibodies. We also thank Pablo Radicella for their critical and careful reading of the manuscript. We also appreciate the help of Elisabetta Andermarcher with the English editing.”

The authors apologize for any inconvenience caused and state that the scientific conclusions are unaffected. The academic editor has checked this correction. These changes do not affect the conclusions presented in the paper. The original article has been updated.
