# Supplementary material for: Rad52 Oligomeric N-Terminal Domain Stabilizes Rad51 Nucleoprotein Filaments and Contributes to Their Protection against Srs2
Source: Cells. 2021 Jun 11;10(6):1467. doi: 10.3390/cells10061467 (PMC8230603; doi:10.3390/cells10061467)
Supplement: Supplementary file 1 [file cells-10-01467-s001.zip › cells-1250041-supplementary.pdf]

Table S1. ***S. cerevisiae*** **strains.**

Strain	Genotype	Source	Strain background*
FF18733	*MAT***a** *leu2-3, 112 trp1-289 ura3-52 lys1-1 his7-2*	F. Fabre	
FF18744	*MAT***a** *srs2::LEU2*	F. Fabre	
FF18742	*MAT***a** *rad52::URA3*	F. Fabre	
ECS2136	*MAT***a** *rad52::URA3 srs2::NAT*	This study	
FF18964	*MAT***a** *rad50::URA3*	F. Fabre	
FF18973	*MAT***a** *rad54::LEU2*	F. Fabre	
FF181495	*MAT***a** *sgs1::URA3*	F. Fabre	
ECS57	*MAT***a** *rrm3::NATMX*	This study	
ECS74	*MAT***a** *mrc1::NATMX*	This study	
ECS75	*MAT***a** *ctf18::NATMX*	This study	
ECS2928	*MAT***a** *rad51::URA3 YCplac111-RAD52-6H-FFF*	This study	
ECS3159	*MAT***a** *rad52::URA3 srs2::NAT YCplac111-RAD52-V95I-6H-FFF*	This study	
ECS3161	*MAT***a** *rad52::URA3 srs2::NAT YCplac111-RAD52-V129A-6H-FFF*	This study	
ECS3163	*MAT***a** *rad52::URA3 srs2::NAT YCplac111-RAD52-D79N-6H-FFF*	This study	
ECS3416	*MAT***a** *rad52::URA3 srs2::NAT YCplac111-RAD52-V95D-6H-FFF*	This study	
ECS3418	*MAT***a** *rad52::URA3 srs2::NAT YCplac111-RAD52-V95F-6H-FFF*	This study	
ECS3420	*MAT***a** *rad52::URA3 srs2::NAT YCplac111-RAD52-V95T-6H-FFF*	This study	
ECS2153	*MAT***a** *rad52::URA3 srs2::NAT YCplcac111*	This study	
ECS3031	*MAT***a** *rad52::URA3 srs2::NAT YCplac111-RAD52-6H-FFF*	This study	
ECS3138	*MAT***a** *rad52::URA3 YCplac111-rad52-V95I-6H-FFF*	This study	
ECS3140	*MAT***a** *rad52::URA3 YCplac111-rad52-V129A-6H-FFF*	This study	
ECS3142	*MAT***a** *rad52::URA3 YCplac111-rad52-D79N-6H-FFF*	This study	
ECS2958	*MAT***a** *rad52::URA3 YCplac111-rad52-R37A-6H-FFF*	This study	
ECS2917	*MAT***a** *rad52::URA3 YCplac111*	This study	
ECS2947	*MAT***a** *rad52::URA3 YCplac111-RAD52-6H-FFF*	This study	

ECS3410	*MAT***a** *rad52::URA3 YCplac111-rad52-V95D-6H-FFF*	This study	
ECS3412	*MAT***a** *rad52::URA3 YCplac111-rad52-V95F-6H-FFF*	This study	
ECS3414	*MAT***a** *rad52::URA3 YCplac111-rad52-V95T-6H-FFF*	This study	
ECS3284	*MAT***a** *rad52::URA3 rad59-9MYC-KAN YCplac111-rad52-6H-FFF*	This study	
ECS3286	*MAT***a** *rad52::URA3 rad59-9MYC-KAN YCplac111-rad52-V95I-6H-FFF*	This study	
ECS3288	*MAT***a** *rad52::URA3 rad59-9MYC-KAN YCplac111*	This study	
ECS2142	*MAT***a** *Rad59::KANMX srs2 ::LEU2*	This study	
FF181770	*MAT***a** *Rad59 ::KANMX*	F. Fabre	
L317.4a	*MAT***a** *arg4-RV*	This study	
L318.1d	*MATα arg4-Bg*	This study	
L317.17c	*MAT***a** *arg4-RV srs2::LEU2*	This study	
L318.7a	*MATα arg4-Bg srs2::LEU2*	This study	
ECS3230	*MAT***a** *arg4-RV rad52-V95I*	This study	
ECS3236	*MATα arg4-Bg rad52-V95I*	This study	
ECS3231	*MAT***a** *arg4-RV rad52-V95I srs2::LEU2*	This study	
ECS3235	MATα *arg4-Bg rad52-V95I srs2::LEU2*	This study	
ECS3239	*MAT***a** *arg4-RV rad52-D79N*	This study	
ECS3245	*MATα arg4-Bg rad52- D79N*	This study	
ECS3241	*MAT***a** *arg4-RV rad52- D79N srs2::LEU2*	This study	
ECS3243	MATα *arg4-Bg rad52- D79N srs2::LEU2*	This study	
EMY110	*ade1-100 ura3-52 leu2-3,112 lys5 hml::ADE1 mat::hisG hmr::ADE1 leu2-cs his4::NAT-leu2Δ5' ade3::GAL::HO RAD52-6H-FFF-KANMX*	J. Haber	YFP17
ECS3220	*srs2::HPH*	This study	
EMY424	*rad52-D79N*	This study	
EMY425	*rad52- D79N srs2::HPH*	This study	
EMY419	*rad52-V95I*	This study	
EMY420	*rad52- V95I srs2::HPH*	This study	
EMY430	*rad52-V95I-6H-FFF*	This study	
EMY441	*rad52- V95I-6H-FFF srs2::HPH*	This study	

EMY443	*rad52-R37A-6H-FFF*	This study	
EMY444	*rad52-R37A-6H-FFF srs2Δ*	This study	
tGI354	*hml::ADE1 MATalpha hmr::ADE1 arg5,6::MATa-inc::HPH1 ade3::GAL::HO*	J. Haber	*JKM146*
ECS2632	*srs2::NAT*	This study	
EMY421	*rad52-V95I*	This study	
EMY422	*rad52- V95I srs2::NAT*	This study	
EMY427	*rad52-D79N*	This study	
EMY428	*rad52- D79N srs2::NAT*	This study	
CTY10-5d	*MAT***a** *ade2 trp1-901 leu2-3,112 his3-200 gal4 gal80 - URA3::lexAop-lacZ*	S. Fields	CTY10-5d
TLM285	*Rad52::KANMX*	This study	

Strains are grouped according to their background, indicated on the first line of each group. Only differences from this genotype are noted subsequently.

* When different from FF18733.

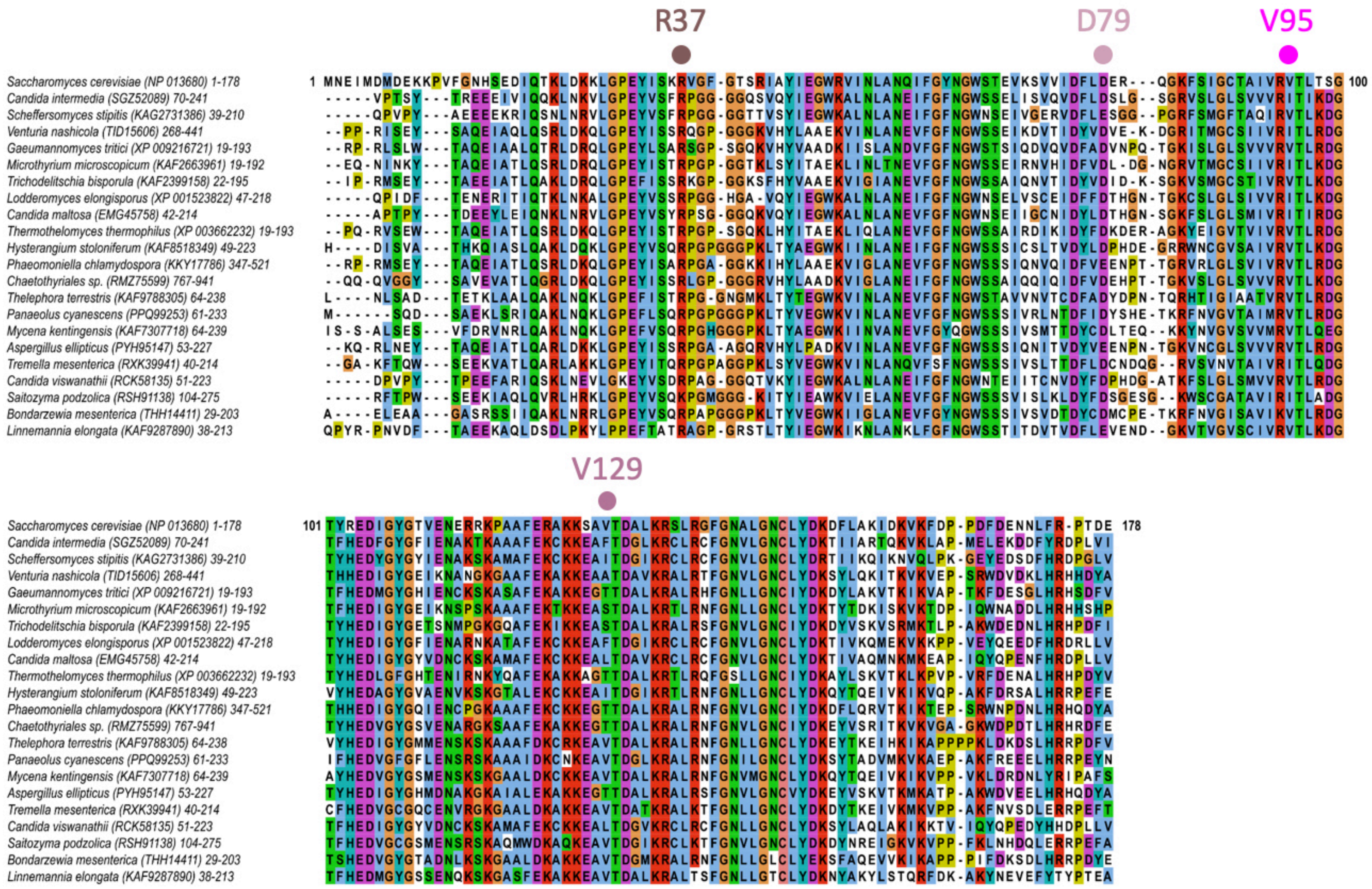

Figure S1. Multiple sequence alignment of a set of 22 representative homologs of *S. cerevisiae* Rad52 (shown on top) restricted to the N-terminal domain region (residues 1-178). The accession number of every homolog is indicated in brackets in the sequence description. The amino acid colors refer to the chemical character of the residuesOn top of the alignment the positions of the mutated residues is indicated by a colored circle.

Table S2. Rad52 mutations that suppress Srs2-deficient cells MMS sensitivity

**Nucleotide change**	**Triplet Change**	**Amino acid change**
G235A	GAT->AAT	D79N
G283A	GTT->ATT	V95I
T386C	GTT->GCT	V129A

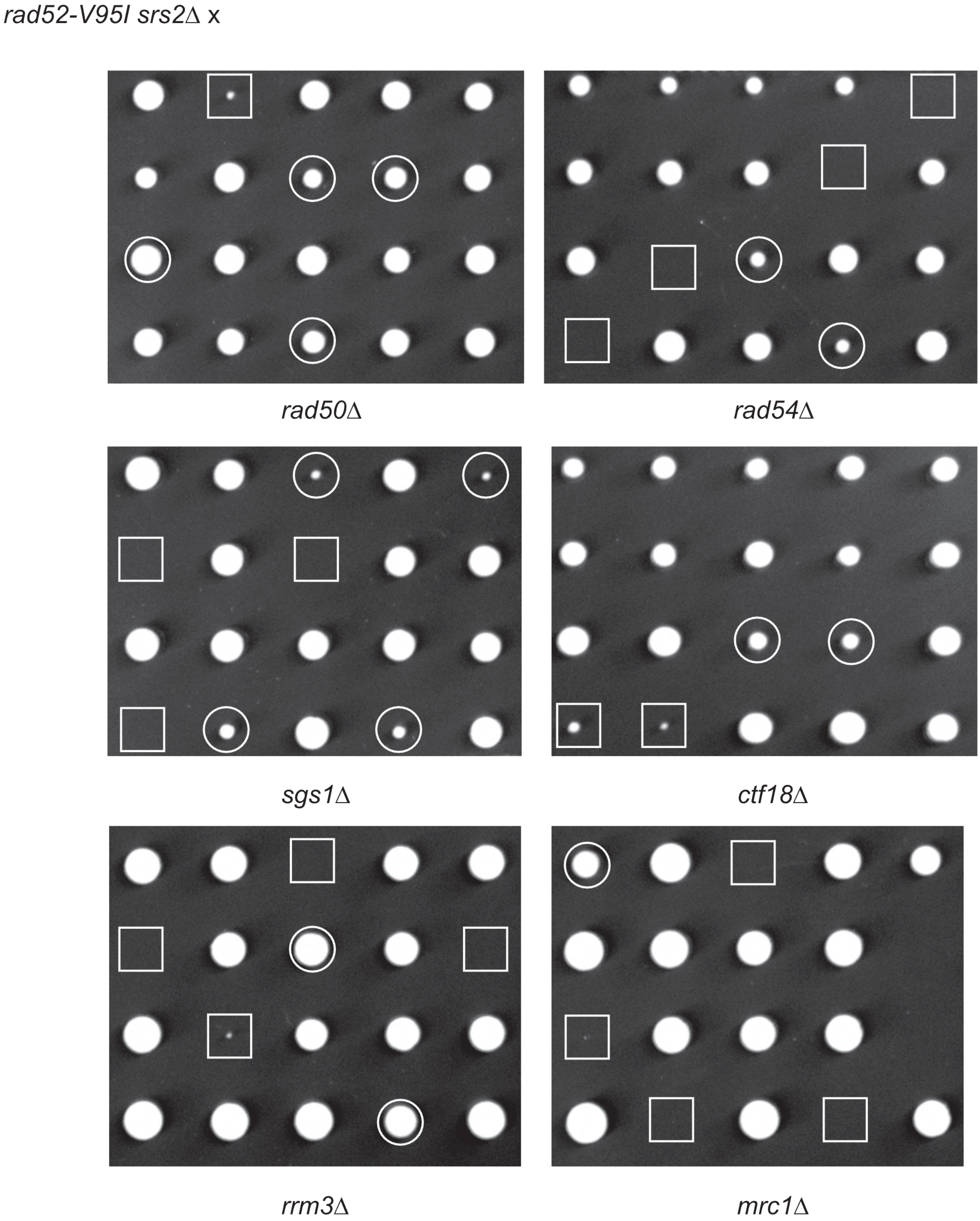

Figure S2. The *rad52-V95I* mutation suppresses the effect of mutations that are synthetically lethal with *srs2*Δ**.** Tetrad analysis of crosses between haploid *rad52-V95I srs2*Δ strains and haploid mutants that are synthetically lethal with *srs2*Δ. Double mutant spores, which do not contain *rad52-V95I*, are indicated by white squares. The white circles mark viable triple mutants.

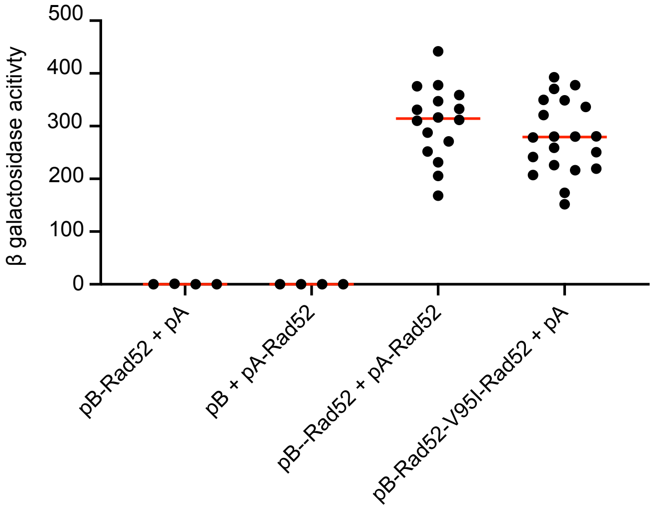

Figure S3. Quantitative measurement of the impact of V95I on Rad52-self-interaction by Y2H analyses. Experiments were done in *rad52*Δ cells to avoid the reduction of the Y2H signal by untagged Rad52. Median β-galactosidase activity is indicated (red line). Controls with empty vectors pBTM116 (pB) or pACT2 (pA) are also shown. The V95I mutation does not affect Rad52-self-interaction.

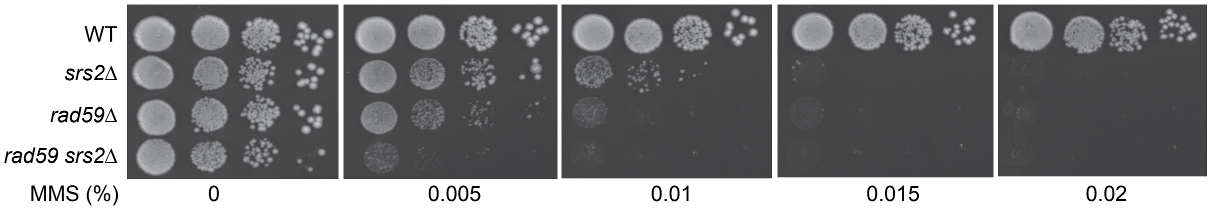

Figure S4. Serial 10-fold dilutions of haploid strains with the indicated genotypes were spotted onto rich media (YPD) containing different MMS concentrations.

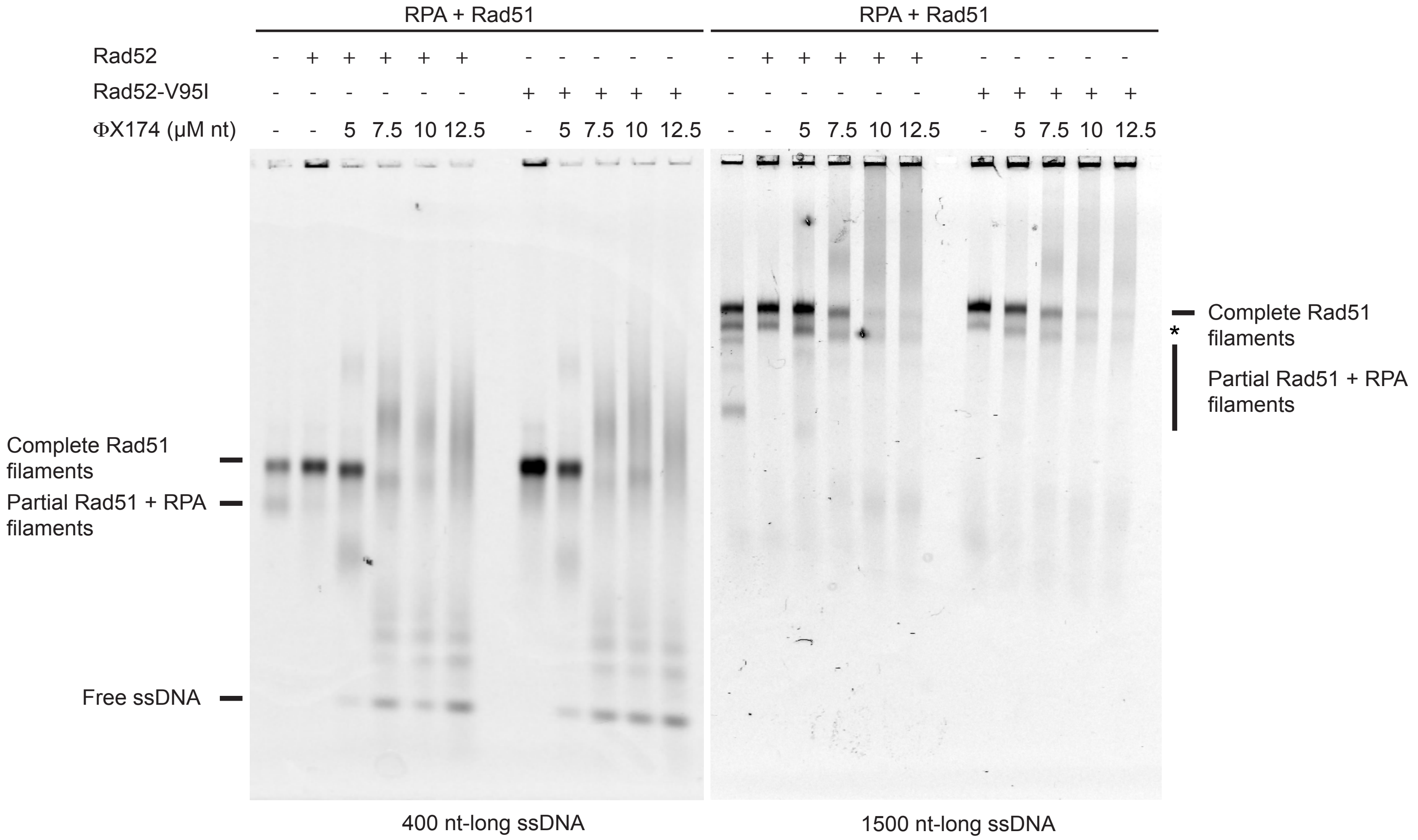

Figure S5.. Rad51 filament stability challenged with excess of ssDNA. All experiments were performed with FLAG-tagged Rad52 proteins. The addition of Rad52 or Rad52-V95I together with Rad51 to RPA-coated 400-nt-long ssDNA or 1500-nt-long ssDNA strongly catalyzes the formation of complete Rad51 filaments. Addition of increasing amounts of competitor ΦX174 ssDNA after Rad51 filament formation leads to their destabilization. Note the formation of an uncharacterized band with 1500-nt-long ssDNA (*).
